# Fecal Microbiota Transplantation Shows Marked Shifts in the Multi-Omic Profiles of Porcine Post-weaning Diarrhea

**DOI:** 10.3389/fmicb.2021.619460

**Published:** 2021-02-23

**Authors:** Yuan Su, Xiaolei Li, Diyan Li, Jing Sun

**Affiliations:** ^1^Animal Genetic Resources Exploration and Innovation Key Laboratory of Sichuan Province, Sichuan Agricultural University, Chengdu, China; ^2^Chongqing Academy of Animal Sciences, Chongqing, China; ^3^Key Laboratory of Pig Industry Sciences, Ministry of Agriculture, Chongqing, China; ^4^Chongqing Key Laboratory of Pig Industry Sciences, Chongqing, China

**Keywords:** post-weaning diarrhea, fecal microbiota transplantation, microbiota, transcriptome, metabolites

## Abstract

Weaning is the most critical phase in pig production and is generally associated with significant impacts on intestinal morphology, structure, physiology, and immune responses, which can lead to subsequent production inefficiencies such as decreases in growth and intake and increases in morbidity and mortality. In the present study, we attempted to explore the effects of fecal microbiota transplantation (FMT) on the fecal microbiota, fecal metabolites, and transcriptome in the jejunum, colon, liver, spleen, and oral mucosa in piglets with post-weaning diarrhea and to evaluate the therapeutic potential of FMT in piglets with post-weaning diarrhea. We found that FMT partially relieved the symptoms of diarrhea in piglets, and microbiota analysis results indicated that *unclassified_f_Prevotellaceae* was identified as an FMT-associated bacterial family at 66 day and that the Shannon index in the healthy group at 34, 38, and 66 days were higher than that at 21 day. Functional enrichment analysis of the oral mucosa, liver, jejunum, and colon showed that most of the differentially expressed genes (DEGs) were enriched in the terms metabolic process, immune response, and inflammatory response. Moreover, the enriched fecal metabolites focused mostly on apoptosis, beta-alanine metabolism, glutathione metabolism, and sphingolipid metabolism. We tried to detect specific “metabolite-bacterium” pairs, such as “*g_Catenisphaera*-stigmastentriol,” “p_Bacteroidetes-(6beta,22E)-6-hydroxystigmasta-4,22-dien-3-one,” and “*g_Prevotellaceae_NK3B31_group*-stenocereol.” Overall, the present study provides a theoretical basis for the alleviation of weaning stress and contributes to the realization of effective and sustainable application of FMT in the pig production industry in the future.

## Introduction

In intensive production systems, piglets are usually weaned at an early age of 3–4 weeks. Weaning is the most critical phase in pig production and is generally associated with a significant impact on intestinal morphology, structure, physiology, and immune responses, which can lead to subsequent production inefficiencies such as decreases in growth and intake and increases in morbidity and mortality (Campbell et al., 2013). In piglet models, the gut microbiota plays an important role in the life of the host (McFall-Ngai et al., 2013; Pluske et al., 2018). Moreover, early weaning of piglets is often accompanied by diarrhea due to gut microbiota interference (Lalles et al., 2007). The diversity of the intestinal microbiota decreases during the weaning transition (Rist et al., 2013; Guevarra et al., 2018), which increases pathogenic bacteria exploit microbiota-derived sources of carbon and nitrogen as nutrients and regulatory signals to promote their own growth and virulence, eliciting inflammation and strikingly increasing the risk of post-weaning diarrhea and enteric infections (Baumler and Sperandio, 2016; Fouhse et al., 2016).

Fecal microbiota transplantation (FMT) is a technique for transplanting the fecal microbiota from a healthy individual into the gut of a recipient individual (Youngster et al., 2014; Lin et al., 2018). As an important therapy for intestinal diseases, including inflammatory bowel disease and irritable bowel syndrome (Borody and Khoruts, 2011; Pigneur and Sokol, 2016; Heath et al., 2018), FMT has attracted some researchers’ attention due to its ability to restore the intestinal microbiota (Cheng et al., 2018; Hu et al., 2018; Xiang et al., 2020). Over the past decade, FMT has been developed rapidly from initial methods that proposed the use of fresh stool to the use of purified and cryopreserved standardized preparations of fecal microbiota from highly selected donors (Staley et al., 2017; Reygner et al., 2020; Tang et al., 2020). A few studies have reported that clinical applications of FMT using encapsulated frozen stool have provided high cure rates in patients with intestinal dysbiosis-related diseases (Smits et al., 2013; Youngster et al., 2014; Kao et al., 2017; Cheminet et al., 2018). In addition, a recent study reported that treatment with capsulized FMT ameliorates post-weaning diarrhea by increasing the relative abundances of Firmicutes, Euryarchaeota, Tenericutes, Lactobacillus, and Methanobrevibacter in the colon of recipient piglets (Tang et al., 2020). Thus, FMT could provide a potential method in which re-establishment of the intestinal microbiota results in diarrhea amelioration in post-weaning piglets.

Currently, with the continuous development of high-throughput sequencing technology, multi-omic has become a necessary collection of different methods to analyze biological problems comprehensively and precisely. A previous study of the effects of early intervention with maternal fecal microbiota and antibiotics on the intestinal microbiota and metabolite profile indicated that maternal FMT markedly influenced the relative abundances of *Clostridium sensu stricto* and *Parabacteroides* in the colon and colonic metabolic profiles in newborn piglets on day 7 (Lin et al., 2018). Many studies have indicated that the weaning transition activates inflammation and stress signaling pathways and leads to abnormal expression of intestinal genes and proteins in pigs (Wang et al., 2008; Hu et al., 2013). Meng et al. (2020) performed RNA-seq to determine the changes in the intestinal transcriptome and conducted 16S rRNA sequencing to measure the gut microbiota changes during the weaning transition. They found that the weaning transition altered the intestinal expression of genes involved in nutrient absorption, transportation, and metabolic processes and significantly decreased the abundances of *Proteobacteria* and *Fusobacteria* in piglets. However, there is a lack of multi-omic characterization of the intestinal microbiota and metabolites and RNA-seq analysis in piglets under post-weaning diarrhea conditions. Early intervention with the intestinal microbiota of piglets by FMT can reduce the diarrhea rate in piglets and play a role in the prevention of diarrhea (Lin et al., 2018). A related question is whether FMT can relieve or treat diarrhea in affected piglets. Currently, there are few reports on the treatment of post-weaning piglets with diarrhea using FMT. To study this topic, we attempted to explore the effects of FMT on the fecal microbiota, fecal metabolites, and transcriptome of the jejunum, colon, liver, spleen, and oral mucosa in piglets with post-weaning diarrhea and to evaluate the therapeutic potential of FMT in piglets with post-weaning diarrhea. The present study provides a theoretical basis for the alleviation of weaning stress and contributes to the realization of effective and sustainable application of FMT in the pig production industry in the future.

## Materials and Methods

### Experimental Design and FMT Experiments

**FIGURE 1:**
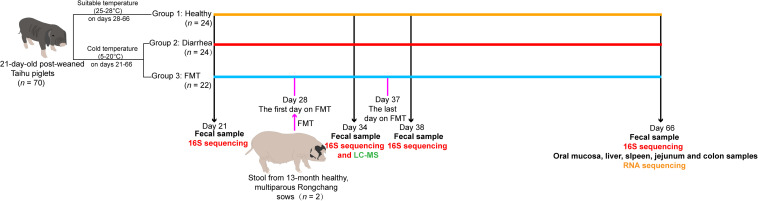
Overview of the workflow for integrated analysis of the gut microbiota, fecal metabolome, and transcriptomes of five tissue types in piglets in different age groups.

All piglets in this study were obtained from Chongqing National Modern Animal Husbandry Demonstration Zone Experimental Pig Engineering Center (Rongchang, Chongqing, China). The whole experimental workflow is summarized in Figure 1. Seventy Taihu piglets with similar body weights were early weaned at 21 day, and all piglets had free access to food and water. We randomly selected 35 piglets to transfer to a separate experimental pig house A; and selected another 35 piglets to transfer to another separate experiment pig house B. The two pig houses were reared in separate pens (three piglets per pen), and were fed enclosures at a natural ambient temperature (5–20°C). After 7 days (from days 21 to 27), different degrees of diarrhea were observed in 46 piglets in experimental pig houses; while the remaining 24 post-weaning healthy piglets (the healthy group) were housed at a suitable temperature (25–28°C), and no diarrhea was observed at 21–65 days. The piglets with diarrhea were randomly allocated to two groups (*n* = 22 for the FMT group; *n* = 24 for the diarrhea group), and detailed experimental treatments were administered as follows: piglets in the FMT group received a fecal microbiota “micro-capsule” in the diet from 28 to 37 day; piglets in the diarrhea group and healthy group received a normal diet. Finally, we randomly selected five piglets from the healthy, FMT and diarrhea groups for sample collection at the corresponding time points. At 21, 34, 38, and 66 days, we collected fecal samples from five piglets per group (the healthy, FMT and diarrhea groups). At 66 day, 15 piglets were euthanized under isoflurane anesthesia, and the oral mucosa, liver, spleen, jejunum, and colon of all piglets were collected. In brief, feces and tissue samples were collected from five biological replicates per group, except for spleen tissue in the FMT group, which was collected from three replicates. Sixty feces and 73 tissue samples were flash frozen in liquid nitrogen and were then stored at −80°C until DNA or RNA was extracted.

Fresh fecal samples used for the preparation of freeze-dried feces for FMT experiments were collected from 13-month-old healthy, multiparous Rongchang sows. As a fecal donor, the two sows need to meet the following conditions: firstly, no clinical symptoms of diarrhea and other major swine diseases, no antibiotic use within 30 days before fecal collection; Secondly, the feces of donor sows were collected and submitted to Suzhou Xishan Biotechnology Co., Ltd. to screen a variety of common intestinal pathogens. Nine screening pathogens (the specific pathogen including *Salmonella spp.*, *Brucella spp.*, *Serpulina hyodysenteriae*, *Actinobacillus pleuropneumoniae*, *myco.*, *Streptococcus suis* type 2, *Staphylococcus aureus*, *Haemophilus parasuis*, and *Erysipelothrix rhusiopathiae*) are all negative in the donor sows, so they can be used as qualified fecal donor sows for making freeze-dried feces which were prepared as described by Staley et al. (2017) with modifications. In brief, 10 g of fresh feces was added to 60 mL of pure water and stirred to homogenize, the homogenate was then filtered through four layers of gauze to obtain the fecal suspension, and sterilized glycerine was added at a 1:9 ratio and mixed well at −20°C for storage. A 2% sodium alginate solution was heated on a magnetic bead agitator to 70°C and stirred, fully dissolved and cooled to room temperature; the fecal suspension and sodium alginate solution were then mixed 1:1 in a beaker. The uniform solution of the fecal suspension and sodium alginate was pumped into a matching syringe and assembled on a uniform granulator, and a large beaker containing a 2% calcium chloride solution was placed directly below the droplet outlet. The uniform granulator was started so that the uniform liquid was squeezed as round droplets into the calcium chloride solution, waiting for all the uniform solution in the syringe to be dripped, resting for 30 min, and waiting for the sodium alginate and calcium chloride to react fully outside the droplet to form an insoluble shell so that the droplet was formed into a granular fecal microbiota micro-capsule; Then, a perforated spoon was used to strain the excess water from the fecal microbiota micro-capsule, which was then packed in a plate and frozen in a freezer at −20°C. When a fecal microbiota micro-capsule was made from the same batch of sub-uniform liquid, the plate containing the fecal microbiota micro-capsule was put into a vacuum freeze dryer and freeze dried at −50°C for approximately 24 h to form small dried particles. Finally, the freeze-dried fecal microbiota micro-capsule was removed and transferred into an aseptic storage bag, which was sealed with a sealing machine. At this point, the fecal microbiota micro-capsule was made and was stored at −80°C. Each piglet in the FMT group was administered a 2-g fecal microbiota micro-capsule daily in the morning for the full experimental period (from days 28 to 37). The recipient piglets in the FMT group were treated continuously from days 28 to 37 by mixing fecal microbiota micro-capsules into a small amount of feed for the recipient piglets.

### Diarrhea Incidence and Diarrhea Index Measurement

The diarrhea index was scored as follows: 0, normal feces (solid); 1, moist feces (semi-solid); 3, loose feces (mild diarrhea); or 5, watery feces (severe diarrhea). Mild diarrhea and severe diarrhea were both considered diarrhea. The diarrhea incidence and index of every piglet were calculated as follows:

Diarrheaincidence(%)=A/M×100%

where A = the total number of days with diarrhea for each piglet. M = the number of days in the whole experimental period.

The diarrhea index was calculated as follows:

Diarrheaindex=B/M×100%

where B = the total score of each piglet and M = the number of days in the whole experimental period.

The diarrhea incidence and index of all piglets in the two groups (FMT and diarrhea group) were calculated by determining the mean values for five piglets per group.

### Microbial Genomic DNA Extraction, 16S rRNA Gene Sequencing and Sequencing Analysis

Microbial DNA was extracted from fecal samples using a QIAamp DNA Stool Mini Kit (Qiagen, GmbH Hilden, Germany) according to the manufacturer’s protocols. DNA integrity was determined by 1% agarose gel electrophoresis, and DNA was quantified using a NanoDrop2000 spectrophotometer (Thermo Fisher Scientific, Wilmington, DE, United States). The V3–V4 hypervariable regions of the bacterial 16S rRNA gene were amplified with primers 338F (5′-ACTCCTACGGGAGGCAGCAG-3′) and 806R (5′-GGACTACHVGGGTWTCTAAT-3′) in a thermocycler PCR system (GeneAmp 9700, ABI, United States). PCR was conducted using the following program: 3 min of denaturation at 95°C; 25 cycles of denaturation for 30 s at 95°C, annealing for 30 s at 55°C, and elongation for 45 s at 72°C; and a final extension step for 10 min at 72°C. PCR was performed in triplicate in a 20 μL mixture containing 4 μL of 5× FastPfu Buffer, 2 μL of dNTPs (2.5 mM), 0.8 μL of each primer (5 μM), 0.4 μL of FastPfu Polymerase, and 10 ng of template DNA. The resulting PCR products were extracted from a 2% agarose gel and further purified using an AxyPrep DNA Gel Extraction Kit (Axygen Biosciences, Union City, CA, United States) and quantified using a QuantiFluor^TM^-ST fluorometer (Promega, United States) according to the manufacturer’s protocol. Purified amplicons were pooled in equimolar amounts, and 2 × 300 bp paired-end sequencing was performed on an Illumina MiSeq platform at Shanghai Majorbio Bio-pharm Technology Co., Ltd. (Shanghai, China).

The raw data obtained by sequencing might contain a certain proportion of interference data, which were first filtered to obtain clean data. Operational taxonomic units (OTUs) were clustered with a 97% similarity cutoff using UPARSE (Edgar, 2013), and chimeric sequences were identified and removed using UCHIME (Edgar et al., 2011; Haas et al., 2011). Sequences with ≥97% similarity were assigned to the same OTU. Then, the SILVA database (Quast et al., 2013) was used based on the Mothur algorithm to annotate taxonomic information for each representative sequence. Subsequently, bioinformatic analysis was performed based on this output normalized data.

Alpha diversity indices (Shannon index) were calculated using the Vegan package in R software and compared by using the Wilcoxon rank sum test (Dixon, 2003). Principal coordinates analysis (PCoA) based on Bray-Curtis distances (Bray and Curtis, 1957) was performed using the ggplot2 package. The different groups were statistically compared using ANOSIM (Chapman and Underwood, 1999) with 10,000 permutations based on the Bray-Curtis ordination to evaluate the rationality of the group divisions. The high-dimensional biomarkers were discovered by LEfSe [linear discriminant analysis (LDA) effect size] with the parameter “LDA score >4” (Segata et al., 2011) to determine the most discriminating taxa.

### Transcriptome Sequencing and Differential Expression Analysis

Total RNA was extracted using an RNeasy Mini Kit (Qiagen, Hilden, Netherlands) following the manufacturer’s instructions. The integrity of total RNA was assessed using a Bioanalyzer 2100 system (Agilent Technologies, Palo Alto, CA, United States) with an RNA 6000 Nano Kit. A total of 3 μg RNA per sample was used as input material for the construction of sequencing libraries. Libraries were generated using an NEBNext Ultra RNA Library Prep Kit for Illumina (NEB, United States) following the manufacturer’s instructions, and index codes were added to connect sequences to samples. Subsequently, a total of 73 sequencing libraries were prepared and then sequenced on the Illumina HiSeq × Ten platform (Illumina Inc., San Diego, CA, United States) at Novogene Bioinformatics Technology Co., Ltd. (Beijing, China) with a paired-end sequencing length of 150 bp.

We removed low-quality reads, including those with low-quality bases, adaptor sequences and poly-N contaminants, and obtained high-quality clean reads. Clean reads were then mapped to the pig reference genome (*Sus scrofa* 11.1)^1^ via Top Hat with the default parameters (Ghosh and Chan, 2016). Gene expression levels were calculated as fragments per kilobase million (FPKM) mapped reads values using HTSeq (Anders et al., 2015). The differentially expressed genes (DEGs) were identified using the DEGSeq package (Wang et al., 2010), and we considered genes with a false discovery rate (FDR) of <0.05 and a | log_2_ fold change| of >1 as significant DEGs. The DEGs were subjected to functional enrichment analysis with Gene Ontology (GO) terms, including molecular function, cellular component, and biological process terms, as well as Kyoto Encyclopedia of Genes and Genomes (KEGG) pathway categories; these analyses were performed with the Metascape online tool (Zhou et al., 2019). The GO terms and KEGG pathways with a *P*-value of <0.01 were considered to be significantly enriched.

### qRT-PCR Validation of RNA-Seq Analysis Results

To validate the repeatability of the RNA-seq analysis results, 13 candidate genes were randomly selected and evaluated by using quantitative real-time PCR (qRT-PCR) (for the primers, see Supplementary Table 1). We performed qRT-PCR in a CFX96 Real-Time PCR Detection system (Bio-Rad Co., Hercules, CA, United States) and detected RNA expression using SYBR Green Real-Time PCR Master Mix (Takara Co., Dalian, China). The relative expression levels were calculated using the 2^–ΔΔCt^ method and normalized to those of the reference gene *GAPDH*.

### Metabolomic Analysis Based on LC/MS

The metabolome measurement and pretreatment protocols were based on the protocols followed by Majorbio Bio-Pharm Technology Co., Ltd. (Shanghai, China), and the detailed steps were consistent with those in Wang’s published article (Wang et al., 2019). LC-MS was performed in an AB SCIEX TripleTOF 5600^TM^ mass spectrometry system (AB SCIEX, United States). Bioinformatic analyses, including PCA and OPLS-DA analysis, performed in this study were conducted on the Majorbio Cloud Platform^2^. Variable importance in projection (VIP) coefficients was calculated in the OPLS-DA model. Differential metabolites statistically significant among groups were selected as those with a VIP value of >1 and a *P*-value of <0.05. Differential metabolites were mapped to their biochemical pathways through metabolic enrichment and pathway analysis based on a database search (KEGG)^3^. Only pathways with an FDR-corrected *P*-value of <0.05 were represented.

### Association Analysis

For correlation analysis, the associations between the abundances of the specified bacteria and significantly differential metabolites were evaluated. Pearson’s *r* values and *P*-values were calculated using the cor.test function, and associations were visualized using the pheatmap function in R software.

## Results

### FMT Increases the Survival Rate of Piglets With Post-weaning Diarrhea

**FIGURE 2:**
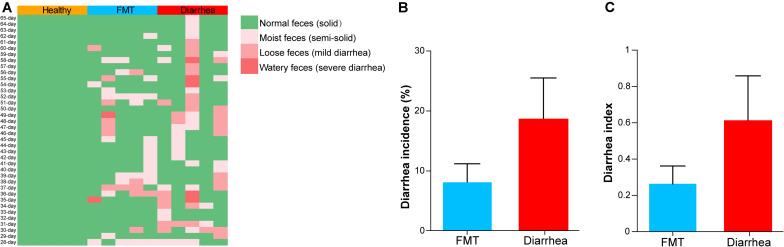
The effects of FMT on the diarrhea incidence and diarrhea index in piglets. **(A)** Fecal morphology and death rate of piglets during the experiment. The mean diarrhea incidence **(B)** and diarrhea index **(C)** of piglets from day 28 to 65 after early weaning. Data are expressed as the mean ± SD (*n* = 5). Statistical analysis was performed with Wilcoxon rank-sum test.

During the experiment (from days 28 to 65), the fecal morphology and death of piglets in the healthy, FMT and diarrhea groups were monitored, and the data are shown in Figure 2A. Interestingly, we found that the average weight gain in the healthy group was significantly higher than that in the FMT and diarrhea groups (*P* < 0.05), whereas there was no remarkable difference measured between the FMT and diarrhea group (*P* > 0.05, Supplementary Table 2). According to the statistics, the diarrhea rate and diarrhea index of piglets in the FMT and diarrhea groups are shown in Figures 2B,C. In addition, we found that the diarrhea rate and diarrhea index of piglets in the FMT group were lower than those of piglets in the diarrhea group from days 28 to 65 (*P* > 0.05). The above results indicated that FMT could partially relieve the symptoms of diarrhea in piglets.

### FMT Alters the Diversity and Structure of Fecal Microbiota in Piglets

**FIGURE 3:**
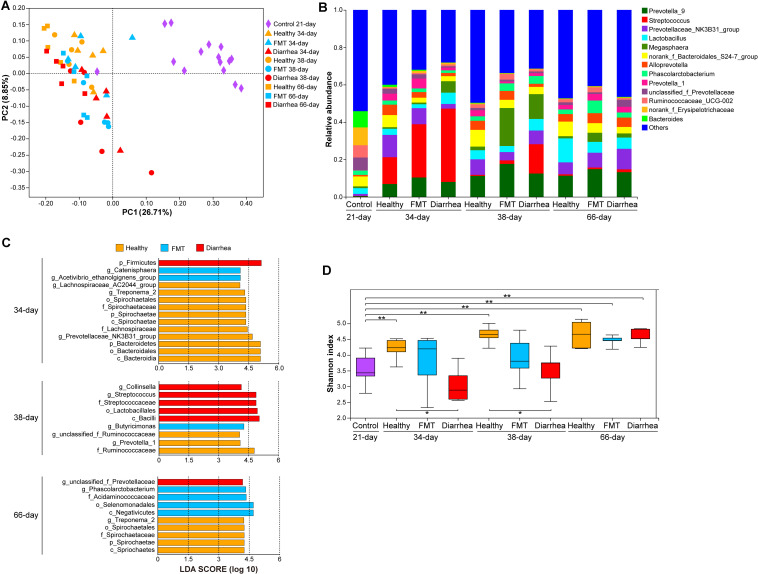
Comparison of the fecal microbiota between piglets in the FMT and diarrhea groups. **(A)** PCoA of the 60 samples based on weighted UniFrac distances. **(B)** The genera with the top 14 relative abundances in the microbiota. **(C)** LEfSe analysis of bacterial taxa showed significant differences among the different groups (LDA score >4). **(D)** Shannon indexes were compared using the Wilcoxon rank-sum test to determine significant differences; *P < 0.05, **P < 0.01.

To describe the fecal microbiota of the piglets, we profiled the taxonomic abundances in 60 samples from 15 piglets in the healthy, FMT and diarrhea groups by sequencing the V3–V4 region of the 16S rRNA gene. A total of 2,367,476 high-quality reads (with an average of 39,458 reads per sample; range, 30,796–74,750) were obtained (Supplementary Table 3). We then used PCoA (Figure 3A) and ANOSIM (Supplementary Table 4) to visualize the differences in the taxonomic composition at the different time points. The taxonomic composition in the diarrhea group was not similar to that in the healthy group at 34 day (*R* = 0.504, *P* = 0.014) or 38 day (*R* = 0.284, *P* = 0.029), while the taxonomic composition in the FMT group was more similar to that in the healthy group; in addition, the taxonomic composition in the FMT group was more similar to that in the diarrhea group (34 day: *R* = −0.060, *P* = 0.708; 38 day: *R* = 0.264, *P* = 0.058).

At the phylum level, similar phyla dominated the microbiota at all four time points (Firmicutes, Bacteroidetes, and Proteobacteria, accounting for 91.57 to 98.26% of the total microbiota) (Supplementary Table 5). At the genus level, *Streptococcus* (14.32–39.24%) was the most abundant genus in all three groups at 34 day; *Prevotella_9* (11.23%), *Megasphaera* (20.32%), and *Streptococcus* (15.70%) dominated the microbiota in the healthy, FMT and diarrhea groups, respectively, at 38 day. At 66 day, the most abundant genus was *Lactobacillus* (12.82%) in the healthy group, while *Prevotella-9* was the dominant genus in the FMT (14.86%) and diarrhea (13.23%) groups (Figure 3B). We then used LEfSe to explore group-associated bacterial features, and these features are visualized in Figure 3C. Firmicutes were classified as a diarrhea-associated bacteria, whereas *Catenisphaera* and *Acetivibrio_ethanolgignens_group* were identified as FMT-associated bacterial genera at 34 day. Notably, *Streptococcaceae* were associated with the diarrhea group, and *Butyricimonas* was identified as an FMT-associated bacterial genus at 38 day. *Unclassified_f_Prevotellaceae* were identified as FMT-associated bacteria at 66 day. Analysis of the Shannon index indicated that the alpha diversity of the microbiota in the healthy group at 34, 38, and 66 days was higher than that in the control group at 21 day (*P* < 0.01), indicating that more microbial taxa were present in this group at these time points than in the control group. Furthermore, the comparisons (healthy vs. diarrhea) at 34 and 38 days showed significant differences (*P* < 0.05, Figure 3D).

### Effects of FMT on the Transcriptomes of Different Tissues in Piglets With Post-weaning Diarrhea

**FIGURE 4:**
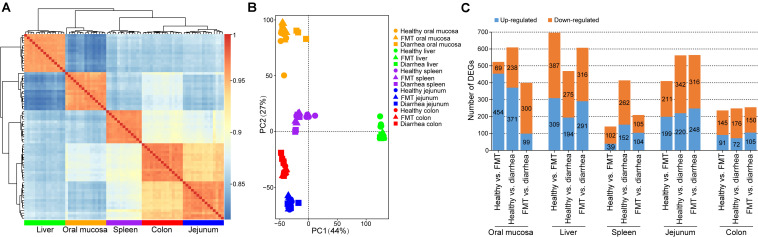
Variation and differential expression of genes in oral mucosa, liver, spleen, jejunum, and colon tissues. **(A)** Spearman’s *r* heatmap of gene expression between every pair of samples. **(B)** Principal component analysis (PCA) plot based on normalized gene expression levels (FPKM). **(C)** Distribution of up- and down-regulated DEGs across all tissues.

To determine the changes in transcript levels due to diarrhea in post-weaning piglets, oral mucosa, liver, spleen, jejunum, and colon tissues were obtained from piglets in the three groups. After filtering out reads containing adaptor sequences and reads with low quality, a total of 607.92 Gb of high-quality reads was obtained, corresponding to an average of ∼8.33 Gb per sample. The high-quality reads were mapped to the pig reference genome (*S. scrofa* 11.1) with a mapping rate of 91.62–95.07% (Supplementary Table 6). Based on the gene expression dynamics, the Spearman correlation between each pair of samples was calculated (Figure 4A). The results showed that the six tissues from each biological replicate were clustered, indicating a tissue-dependent expression pattern. Furthermore, the PCA results showed that there was a high degree of variance between different tissues, which contrasted with the Spearman’s *r* heatmap of gene expression (Figure 4B). To evaluate DEGs in different tissues, we compared the expression levels between different groups with the following threshold criteria: | log_2_ fold change| > 1 and FDR < 0.05. Overall, a larger number of DEGs were identified in the comparisons of the healthy vs. diarrhea group than in the comparisons of the other two group pairs; the oral mucosa and spleen in the FMT vs. diarrhea group comparison had the second highest number of DEGs (Figure 4C and Supplementary Table 7). Similar results were found in the jejunum and colon.

To further investigate the variations in the biological functions of these DEGs across different tissues and different groups, we performed functional enrichment analysis on the DEGs. Functional enrichment analysis of DEGs in the oral mucosa of the healthy vs. diarrhea and FMT vs. diarrhea groups showed that most of these DEGs were enriched in metabolic process, immune response, and inflammatory response terms, such as fatty acid metabolic process (GO:0006631), adaptive immune response (GO:0002250), humoral immune response (GO:0006959), and acute inflammatory response (GO:0048608) (Supplementary Table 8). In addition, the DEGs in the liver in the FMT vs. diarrhea group were significantly enriched in the terms lipid biosynthetic process (GO:0008610), phospholipid metabolic process (GO:0006644), acylglycerol biosynthetic process (GO:0046463), and phospholipid biosynthetic process (GO:0008654) (Supplementary Table 8). Interestingly, comparison of the jejunum and colon between the FMT and diarrhea groups revealed that the DEGs were significantly enriched in the terms production of molecular mediator of immune response (GO:0002440), glucose metabolic process (GO:0006006), and carboxylic acid biosynthetic process (GO:0046394) (Supplementary Table 8).

### qRT-PCR Validation of Gene Expression

A total of 13 DEGs (*S100G*, *FABP6*, *ACAA2*, *SLC1A4*, *GATM*, *CXCL2*, *STAT3*, *CSF3R*, *CXCL14*, *PPAR-*γ, *SIGLEC1*, *CYP2C42*, and *LTF*) in different tissues were randomly selected for validation by qRT-PCR (Table 1). The fold change (2^–ΔΔCt^) values of the 13 DEGs in each sample determined by qRT-PCR was compared with the log_2_ (fold change) values obtained by RNA-seq. Our results demonstrated that the expression patterns of the 13 DEGs in different tissues were generally consistent with the expression patterns identified by RNA-seq, suggesting that the RNA-seq results were accurate and reliable.

**TABLE 1:** Validation of selected RNA-seq-based gene expression data by qRT-PCR.

Tissue	Gene	Healthy vs. FMT		Healthy vs. diarrhea		FMT vs. diarrhea	
		RNA-seq (log_2_ fold change)	qRT-PCR (2^–ΔΔCt^)	RNA-seq (log_2_ fold change)	qRT-PCR (2^–ΔΔCt^)	RNA-seq (log_2_ fold change)	qRT-PCR (2^–ΔΔCt^)
Oral mucosa	*S100G*	3.490	11.924	9.272	11.924	5.782	2.178
	*FABP6*	6.382	1.139	9.461	1.234	3.079	0.923
Liver	*ACAA2*	−0.946	0.397	1.131	3.973	2.077	10.002
	*SLC1A4*	−2.218	0.159	−0.853	0.623	1.366	3.905
	*GATM*	−2.137	0.186	−0.118	0.756	2.020	4.076
Spleen	*CXCL2*	0.243	1.277	0.367	1.414	0.124	1.107
	*STAT3*	0.023	1.166	0.126	1.183	0.103	1.014
	*CSF3R*	−0.766	0.463	−1.098	0.377	−0.332	0.813
	*CXCL14*	0.141	1.242	−0.207	0.872	−0.347	0.702
	*PPAR-*γ	0.453	1.449	0.707	1.694	0.255	1.169
Jejunum	*SIGLEC1*	−0.710	0.548	0.366	1.347	1.076	2.457
	*CYP2C42*	0.715	0.968	1.103	1.588	0.388	1.640
Colon	*SIGLEC1*	−0.885	0.465	−0.164	0.747	0.721	1.606
	*LTF*	−1.403	0.221	−5.565	0.210	−4.162	0.949

*The positive and negative values indicate up- and down-regulation, respectively, in the comparisons. RNA-seq data are shown as log_2_ fold change values, and qRT-PCR data were analyzed by the 2^–ΔΔCt^ method. All measurements of qRT-PCR were performed in triplicate on each of three independent biological replicates.*

### Metabolic Changes in the Recipient Piglets Following FMT

**FIGURE 5:**
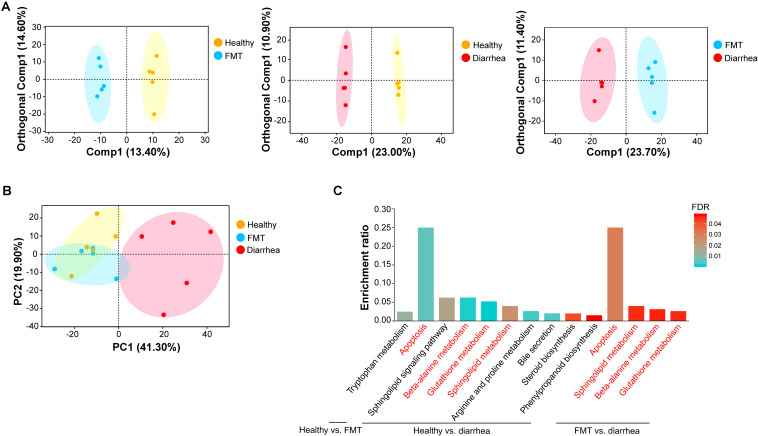
Multivariate statistical analysis of fecal metabolomic and pathway enrichment analysis of significant metabolites. **(A)** OPLS-DA score plots for each pair of groups. **(B)** PCA score plots for fecal samples in the three groups. Each point represents an individual sample. **(C)** Overview of metabolites that were enriched in the three pairwise comparisons. The red font indicates the KEGG metabolic pathways that were enriched in every pairwise comparison.

Next, to investigate the effects of FMT on the fecal metabolome in piglets with diarrhea, we analyzed the fecal metabolome in the three groups. A supervised OPLS-DA pattern recognition method was applied to identify the overall metabolic differences between each pair of groups. As shown in Figure 5A, a significant trend toward separation was observed between every pair of groups. Moreover, PCA analysis revealed good separation of metabolites between the healthy and diarrhea groups. No obvious separation was found between the healthy and FMT groups or between the FMT and diarrhea groups (Figure 5B).

A total of 14 significantly differential metabolites between the healthy and FMT groups were identified using a VIP threshold of 1 (*P* < 0.05), and 3 metabolites [stenocereol, 9(S)-HpODE, and gingerglycolipid C] were classified as lipids and lipid-like molecules (Table 2). Compared with those in the healthy group, the levels of four lipids and lipid-like molecules [including (6beta,22E)-6-hydroxystigmasta-4,22-dien-3-one, ursolic acid, DG(15:0/20:2(11Z,14Z)/0:0), and stenocereol] were significantly decreased in the diarrhea group. Furthermore, regarding the key significant metabolites in the diarrhea group, the levels other five lipids and lipid-like molecules except for propyl 2,4-decadienoate were significantly decreased compared to those in the FMT group. KEGG analysis of the significantly differential metabolites was performed to investigate the metabolic mechanisms that were affected by FMT in piglets with diarrhea. The enrichment analysis results (Figure 5C) showed that metabolic pathways such as apoptosis, beta-alanine metabolism, glutathione metabolism, and sphingolipid metabolism were significantly affected (*P* < 0.05) by changes in the healthy vs. diarrhea and FMT vs. diarrhea groups.

**TABLE 2:** Significant metabolites among the three groups.

Pairwise comparison	Metabolite	VIP	Fold change	Trend
Healthy vs. FMT	**Stenocereol**	1.910	0.774	↓*
	**9(S)-hpODE**	1.577	3.235	↑*
	**Gingerglycolipid C**	1.212	2.511	↑*
	Leu-Leu-Leu	1.083	13.313	↑*
	L-histidinol	1.117	1.383	↑*
	(6R,8Z)-6-hydroxy-3-oxotetradecenoic acid	16.112	67.971	↑*
	2,3-dinor prostaglandin E1	3.547	5.079	↑*
	Robenidine	2.679	0.441	↓*
	1,25-dihydroxy-2-nor-1,2-secovitamin D3	1.942	0.193	↓*
	*Cis*-12-Octadecenoic acid methyl ester	1.636	0.062	↓*
	Apicidin	1.490	3.222	↑*
	2,3-dihydrobenzofuran	1.437	0.174	↓*
	13,14-dehydro-15-cyclohexyl carbaprostacyclin	1.255	2.777	↑*
	(22E)-1α-hydroxy-22,23-didehydrovitamin D3	1.006	0.382	↓*
Healthy vs. diarrhea	**(6beta,22E)-6-hydroxystigmasta-4,22-dien-3-one**	5.193	0.593	↓*
	**Ursolic acid**	2.426	0.491	↓*
	**DG(15:0/20:2(11Z,14Z)/0:0)**	1.760	0.327	↓*
	**Stenocereol**	1.671	0.620	↓*
	2-hydroxy-3-methoxyestrone	2.367	0.232	↓*
	Stearoylethanolamide	3.198	2.014	↑*
	MG(18:1(9Z)/0:0/0:0)[rac]	5.513	0.495	↓*
	PG(14:0/21:0)	4.296	0.268	↓*
	1α-hydroxyvitaminD3/1α-hydroxycholecalciferol	2.348	0.552	↓*
	(±)-3′,4′-methylenedioxy-5,7-dimethylepicatechin	1.238	0.336	↓*
	*Cis*-12-octadecenoic acid methyl ester	1.088	0.040	↓*
FMT vs. diarrhea	**(6beta,22E)-6-hydroxystigmasta-4,22-dien-3-one**	4.697	0.657	↓*
	**4,4-dimethylcholesta-8,14,24-trienol**	3.336	0.468	↓*
	**Stigmastentriol**	2.401	0.652	↓*
	**Melilotoside A**	1.870	0.345	↓*
	**Cer(t18:0/16:0)**	1.744	0.598	↓*
	**Propyl 2,4-decadienoate**	1.054	3.683	↑*
	2-hydroxy-3-methoxyestrone	1.821	0.313	↓*
	Ricinoleic Acid methyl ester	5.364	3.469	↑*
	Cer(d16:1(4E)/20:0(2OH))	4.048	2.918	↑*
	PG(14:0/21:0)	3.060	0.460	↓*
	AM3102	2.696	16.373	↑*
	2,3-dinor prostaglandin E1	2.144	0.297	↓*
	Palmitoyl ethanolamide	1.533	1.582	↑*
	Glu-Arg-Glu	1.296	0.306	↓*
	2,3-dihydrobenzofuran	1.046	6.149	↑*

The up (fold change >1) and down (fold change <1) arrows represent the relative increases (↑) and decreases (↓) in the levels of the metabolites, respectively. Significant differences in metabolites (* P < 0.05) between pairs of groups were determined by the Wilcoxon rank sum test. Lipids and lipid-like molecule metabolites are presented in bold font.

### Correlation Analysis of the Specific “Metabolite-Bacterium” Pairs

**FIGURE 6:**
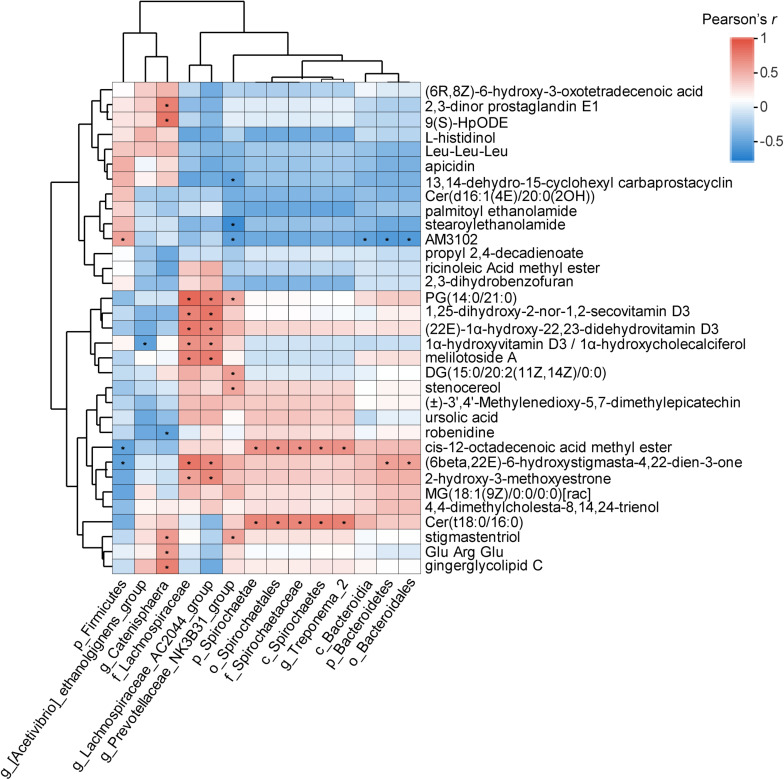
Correlation analysis between the significantly differential metabolites and fecal bacterial taxa identified by LEfSe according to Pearson’s *r* for fecal metabolite types and bacterial taxa (*P < 0.05). Red represents a positive correlation, while blue represents a negative correlation.

Pairwise Pearson correlation analysis was conducted on the 14 bacterial taxa and 33 metabolites (Figure 6). Based on the results, the correlations between 462 of the 46 pairs (9.96%) appeared to be significant (*P* < 0.05). Among these pairs, 36 (78.26%) were positively correlated, and 10 (21.74%) were negatively correlated. *g_Catenisphaera* was positively correlated with stigmastentriol, Glu-Arg-Glu, and gingerglycolipid C; p_Spirochaetae and *g_Treponema_2* were positively correlated with Cer(t18:0/16:0); p_Bacteroidetes was positively correlated with (6beta,22E)-6-hydroxystigmasta-4,22-dien-3-one, while p_Firmicutes was negatively correlated with (6beta,22E)-6-hydroxystigmasta-4,22-dien-3-one. Additionally, *g_Prevotellaceae_NK3B31_group* was positively correlated with stigmastentriol, DG(15:0/20:2(11Z,14Z)/0:0), and stenocereol (Figure 6).

## Discussion

Some recent studies reported that supplementation with probiotics influenced intestinal health, alleviated diarrhea severity, enhanced gut health, and reduced systemic inflammation in weaned pigs (Kim et al., 2019; Luise et al., 2019; He et al., 2020). However, healthy donor feces have a more complete microbial structure and microbial metabolites than probiotics or composite probiotics (van Nood et al., 2014). Previous studies have demonstrated that FMT can effectively relieve diarrhea symptoms associated with a variety of human intestinal diseases and can even cure related intestinal diseases (Borody et al., 1989; Borody and Khoruts, 2011). As expected, the diarrhea rate and diarrhea index of piglets in the FMT group were lower than those of piglets in the diarrhea group from days 28 to 65 (*P* > 0.05) in our experiment. This difference may be because FMT partially reversed the imbalance in the intestinal microbiota of piglets with diarrhea and improved the resistance of these piglets to disease, thus relieving the symptoms of diarrhea in piglets.

The proportions of Firmicutes and Bacteroidetes, the dominant phyla, in the fecal microbiota of piglets at each time point were relatively high, and this result was consistent with previous studies (Hu et al., 2017; Adhikari et al., 2019; Sun et al., 2019). Bacteroidetes and Spirochaetae were biomarkers of healthy piglets at 34 day. Some scholars have demonstrated that the Spirochaetae can decompose cellulose, pectin, and phosphate, and the SCFAs produced by fermenting carbohydrates can not only regulate the immune system function of the host but also provide energy for the animal body (Cummings et al., 1987; Hess et al., 2011). Interestingly, we found that the relative abundance of Spirochaetae in the FMT group was greater than that in the diarrhea group at 34 day. Bacteroidetes can directly regulate intestinal function by regulating the expression of a variety of genes in the host (Freitas et al., 2003), including genes involved in several important intestinal functions, including nutrient absorption, mucosal barrier fortification, xenobiotic metabolism, angiogenesis, and postnatal intestinal maturation (Hooper et al., 2001). The relative abundance of *unclassified_f__Prevotellaceae* in newborn piglets with diarrhea was significantly higher than that in healthy piglets (*P* < 0.05), and this family had a high LDA score, indicating that a high abundance of *unclassified_f__Prevotellaceae* in piglets with diarrhea should be used as an index of susceptibility to post-weaning diarrhea (Dou et al., 2017; Han et al., 2019). Our results found that *unclassified_f_Prevotellaceae* was a FMT-associated bacterial family at 66 day, which is consistent with these previous results. Seekatz et al. (2014) found that FMT can significantly increase the Shannon index of diarrhea patients, making the structure and composition of the recipients’ intestinal microbiota more similar to that of the healthy donors, which provides support for our results. Overall, FMT reversed the decrease in the relative abundance of some beneficial bacteria and increased the abundance of beneficial bacteria in the recipient piglets.

According to our results, functional enrichment analysis in the oral mucosa, liver, jejunum, and colon showed that most of the DEGs (including *MUC*, *PPAR*, and *CHAC1*, etc.) were enriched in the terms metabolic process, immune response, and inflammatory response. We found that FMT affected the expression of *MUC* in the jejunum, indicating that autophagy could be regulated by FMT intervention in the intestinal microbiota of piglets with diarrhea. Mucins (MUC1 and MUC2) produced by intestinal epithelial cells or goblet cells participate in the formation of the mucus layer in the mechanical barrier, which can prevent bacteria from adhering to the intestinal epithelium, translocating, and promoting the clearance of pathogenic bacteria (Turner, 2009). Intestinal mucus defects are also found in various intestinal diseases, such as ulcerative colitis in mice lacking specific *MUC* genes (Heazlewood et al., 2008). *PPAR* plays an important role in maintaining the metabolic dynamic balance in adipose tissue, liver and intestinal tissue, and inflammation occurs easily when metabolism is disrupted (Wahli and Michalik, 2012). The *CHAC1* gene is involved in the regulation of endoplasmic reticulum stress, and some studies have shown that there is a link between endoplasmic reticulum stress and inflammation (Park et al., 2010; Blohmke et al., 2012). Perra et al. (2018) reported that *CHAC1* is highly expressed in patients with excessive respiratory tract inflammation caused by cystic fibrosis, and they speculated that *CHAC1* is involved in the regulation of bronchial inflammation. In this study, the expression of *CHAC1* in the jejunum and colon of piglets in the diarrhea group was higher than that in piglets in the healthy and FMT groups, which indicates that piglets in the diarrhea group had severe intestinal inflammation and up-regulated *CHAC1* expression.

Fecal metabolomic analysis suggested that there were significant differences in the metabolites among the healthy group, FMT group and diarrhea group, which was consistent with the difference in the fecal microbiota among the three groups. Moreover, the significant differences in metabolites among the groups were mainly in lipids and lipid-like molecules, which can be explained by the role of bacteria as regulators for the digestion, absorption, storage, and secretion of dietary lipids in the intestinal tract (Rabot et al., 2010; Sommer and Bäckhed, 2016; Martinez-Guryn et al., 2018). Sphingolipids play an important role in some cellular activities. Sphingomyelin metabolites, especially ceramide (Cer), and sphingomyelin, are key bioactive molecules that regulate cellular functions, such as the cell cycle, senescence, apoptosis, and inflammation (Hannun and Obeid, 2008). Our findings reported that the levels of five metabolites that were lipids or lipid-like molecules, including Cer(t18:0/16:0), were significantly lower in the diarrhea group than in the FMT group. Thus, FMT intervention may play a positive role in suppressing inflammation in recipient piglets with diarrhea. Our metabolite enrichment analysis revealed that apoptosis, beta-alanine metabolism, glutathione metabolism, and sphingolipid metabolism were significantly affected (*P* < 0.05) in the diarrhea group. It has been reported that the apoptotic pathway is affected by abnormal lipid metabolism and impaired intestinal epithelial integrity in piglets with diarrhea (Patwardhan et al., 2016). The change in the intestinal microbiota of weaned piglets is directly related to the change in the intestinal redox state, which makes the differential metabolites significantly enriched in the glutathione metabolic pathway (Patwardhan et al., 2016). Because these metabolites and bacterial taxa may play important roles in piglets with diarrhea via FMT intervention, we tried to detect some specific “metabolite-bacterium” pairs. However, future studies should be conducted on the correlations among these metabolites and bacteria to determine the causes and mechanism of their interaction.

## Conclusion

In summary, our findings suggested that FMT might be a potentially effective approach to reduce the diarrhea rate and modulate the fecal microbiota composition, metabolic processes, and expression of immune-related genes in piglets with post-weaning diarrhea. Thus, the results of this study provide a reference for the treatment of piglets at risk of diarrhea and even other mammalian gut diseases. In addition, further studies are needed to determine the causes and mechanisms of the interactions among specific “metabolite-bacterium” pairs in piglets with post-weaning diarrhea.

## Data Availability Statement

The datasets presented in this study can be found in online repositories. The names of the repository/repositories and accession number(s) can be found below: http://bigd.big.ac.cn/gsa/s/17819Guo, CRA002937 http://bigd.big.ac.cn/gsa/s/x45w2mBO, CRA002947.

## Ethics Statement

The animal study was reviewed and approved by the Institutional Animal Care and Use Committee (IACUC) of Sichuan Agricultural University and Chongqing Academy of Animal Science.

## Author Contributions

DL and JS conceived and designed the study. YS and XL conducted the animal work and most of the laboratory work and wrote the manuscript. All authors performed the bioinformatic analyses and read and approved the final manuscript.

## Conflict of Interest

The authors declare that the research was conducted in the absence of any commercial or financial relationships that could be construed as a potential conflict of interest.

## Footnotes

1 ftp://ftp.ensembl.org/pub/release-91/gtf/sus_scrofa/

2 https://cloud.majorbio.com

3 http://www.genome.jp/kegg/

## Supplementary Material

Supplementary Table 1 Primer sequences used for qRT-PCR.

Supplementary Table 2 The effects of FMT on growth performance in piglets.

Supplementary Table 3 Summary statistics of the 16S rRNA gene sequencing results.

Supplementary Table 4 Analysis of similarity of Bray-Curtis distances.

Supplementary Table 5 Relative abundances of the top 9 components of the microbiota at the four time points at the phylum level.

Supplementary Table 6 Summary statistics of the RNA sequencing results.

Supplementary Table 7 List of DEGs in the pairwise group comparisons.

Supplementary Table 8 Functional categories of DEGs in the pairwise group comparisons.
